# Global response to antibiotic exposure uncovers a critical role for nucleotide metabolism in high-level β-lactam tolerance

**DOI:** 10.21203/rs.3.rs-7829012/v1

**Published:** 2025-10-14

**Authors:** Megan Renee Keller, Misha Iqbal Kazi, Anas Saleh, Upasana Basu, Jung-Ho Shin, Kyu Rhee, Tobias Dörr

**Affiliations:** Cornell University; Cornell University; Weill Cornell Medicine; Cornell University; Cornell University; Weill Cornell Medicine; Cornell University

**Keywords:** Antibiotic Tolerance, Nucleotides, β-lactam, Metabolism, Pentose Phosphate Pathway

## Abstract

Antibiotic tolerance, the ability to survive lethal antibiotics for a prolonged period of time is a rising threat due to its role as a steppingstone towards antibiotic resistance. While tolerance has been recognized as a severe clinical threat, little is known about the mechanisms promoting tolerance. Here, we delineated the physiology of antibiotic tolerance to the classic β-lactam antibiotic, penicillin, to discover the metabolic underpinnings of how tolerant bacteria survive ordinarily lethal antibiotic exposure. We used transcriptomics and metabolomics in the hypertolerant Gram-negative cholera pathogen, *Vibrio cholerae*, to identify the global regulatory and metabolic response to antibiotic exposure. Key pathways like central carbon metabolism, cell wall synthesis, heat shock, two-component systems, and particularly nucleotide synthesis were significantly altered in response to PenG. Most notably, nucleotide levels were depleted upon antibiotic exposure, concomitant with upregulation of both purine and pyrimidine synthesis functions. Consistent with a crucial role for nucleotide synthesis in tolerance, we find that targeting nucleotide synthesis synergizes with penicillin. These datasets thus reveal new vulnerabilities in tolerant bacteria that can serve as conceptual scaffolds for drug development and for improving antibiotic efficacy.

## Introduction

Antibiotic resistance continues to be a severe public health threat^1^. However, antibiotic susceptibility is a spectrum, with multiple steps between full susceptibility and outright resistance. One such steppingstone towards resistance is antibiotic tolerance, or the ability to survive high doses of a typically bactericidal antibiotic for prolonged periods of time ^2–7^. Tolerance has been observed in response to diverse antibiotics, particularly the β-lactams. β-lactam antibiotics are among our most widely prescribed antibiotics, and rates of antibiotic resistance to these crucial drugs are at an all-time high. What makes this class of antibiotics so effective lies in their target: β-lactams inhibit the main enzymes that synthesize the bacterial peptidoglycan layer (penicillin-binding proteins, PBPs). This vital element to the bacterial cell envelope regulates cell shape, turgor pressure, and it is unique to prokaryotes, limiting β-lactam off-target toxicity when used in humans. Bacterial cells that are tolerant to these antibiotics effectively shed their peptidoglycan, forming cell wall-deficient spheroplasts, thereby bypassing cell death; this has been observed in diverse and significant clinical pathogens, including *Enterobacter cloacae, Klebsiella pneumoniae, Pseudomonas aeruginosa, Haemophilus influenzae* and *Acinetobacter baumannii*, and in diverse growth conditions without osmotic stabilization, including standard laboratory media, human serum medium, minimal media, and human patients ^8–13^. Additionally, after the antibiotic is removed from the environment, the cells can revert into their normal rod shape and continue to cause an infection. Data from clinical isolates suggest that this phenotype is pervasive in the clinical setting^10^, and may underlie treatment failure and development of antibiotic resistance. Spheroplasts are metabolically active, but do not replicate in the presence of antibiotic. They rely on cell envelope stress responses to counter the many stresses associated with cell wall loss^10,14–16^. For example, β-lactam antibiotics kill not only by their primary target (inhibiting cell wall synthesis), but also through increasing metabolic burden via a process termed “futile cycling” ^17,18^. Futile cycling is the constant generation of PG strands that are immediately broken down by autolysins. In *E. coli*, this results in poorly-understood perturbances in redox state and enhanced protein synthesis^17^. How tolerant bacteria withstand this metabolic reprogramming is unknown.

Here, we incorporate transcriptomic and metabolomic data to characterize the global antibiotic tolerance response to the cell wall targeting antibiotic, penicillin, in the hypertolerant Gram-negative pathogen *Vibrio cholerae*. We find that central carbon metabolism is rerouted towards cell wall synthesis (depletion of upper glycolysis intermediates), supporting the notion of ongoing futile cycling, which ultimately results in the depletion of nucleotide precursors (likely via reduction of upper glycolysis shuttling into the pentose phosphate pathway). Consistent with an important role for nucleotide homeostasis in β-lactam tolerance, we show that an inhibitor of the pentose phosphate pathway potentiates PenG-mediated killing. Overall, our data demonstrate the complex metabolic perturbances that tolerant cells must manage, showcasing new potential vulnerabilities that could be exploited as targets for new antimicrobial adjuvants.

## Results

### Vibrio cholerae’s global response to penicillin exposure.

**Figure 1:**
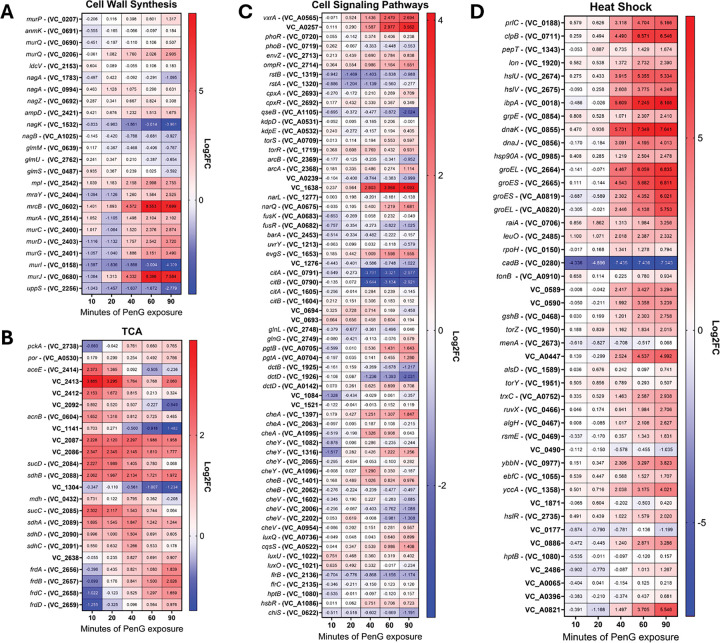
Transcriptomic response to penicillin treatment over time. A) Cell wall synthesis genes. B) Tricarboxylic acid cycle. C) Cell signaling pathways. D) Heat shock response. See methods for details. Cut-off is p-value < 0.05, with raw log2 FC presented.

Using the causative agent of cholera disease, *Vibrio cholerae*, as a model system for investigating tolerance to cell wall-active antibiotics, we first probed global cellular transcript abundance. We treated wild-type N16961 cells with 100μg/mL PenG (10x MIC) ^19^ , and took samples at 0, 10-, 20-, 40-, 60-, and 90-minutes post treatment. This timeframe captures the full process of spheroplast formation as determined previously ^19^. The utility of our tolerance model is that here, unlike most previous studies (in *E. coli*), RNAseq was conducted on intact cells treated extensively with high, supra-MIC concentrations of the antibiotic, whereas in non-tolerant bacteria, sub-MIC concentrations or shorter timeframes had to be used (Fig. 1A–D).

Genes associated with cell wall biosynthesis were upregulated, with only a few genes repressed (Fig. 1A). This was expected, as penicillin treatment was previously shown to upregulate genes involved in maintaining the cell wall, via activation of the VxrAB cell envelope stress response two-component system ^14^. The *nagK* (*vc1532*) gene was one of the few repressed genes, with a marked, 25-fold decrease in expression. NagK is an *N*-acetylglucosamine (GlcNAc) kinase that converts internal GlcNAc to GlcNAc-6P. Internal GlcNAc is most likely the product of peptidoglycan recycling, for *V. cholerae* has no other means of synthesizing unphosphorylated GlcNAc ^20,21^. Genes involved in early cell envelope precursor synthesis also exhibited a (albeit less pronounced) reduction in gene expression, such as the *uppS* (*vc2256*) and *murI* (*vc0158*) genes. UppS is the important undecaprenyl diphosphate synthase whose role is to build the undecaprenyl tether that allows the fundamental PG building block lipid II to traverse the inner membrane into the periplasmic space. MurI functions as a glutamate racemase, converting L-Glu to D-Glu to be incorporated into the growing amino acid side stem of the lipid II molecule ^22^. The downregulation of these genes may reflect a fitness trade-off associated with the generation of their products.

Most genes involved in peptidoglycan synthesis consistently exhibited an increase in gene expression. Genes like *murA, murC, murD, murG*, and *murJ*, which are all involved in the building of the lipid II molecule and flipping it across the inner membrane exhibited a 4- to >20-fold increase in expression. This suggests that penicillin exposure, and its associated cell wall focused damage, triggers the cell to build more PG in order to repair this damage, and/or to prepare for recovery when the antibiotic is removed. The gene coding for aPBP1B, one of the major bifunctional cell wall synthases, *mrcB*, was also highly upregulated. This result acts as an internal control, since we have previously shown that PBP1b is induced by b-lactam exposure through the two-component system VxrAB^16^. This makes physiological sense, as aPBPs are the primary PG synthases involved in cell wall damage repair ^23^.

VxrAB is a well-characterized system for detecting cell-wall stress and subsequent β-lactam tolerance in *V. cholerae* ^16^. The two-component system is localized to the inner membrane, and upon activation by cell wall damage through an unknown signal, the histidine kinase VxrA phosphorylates VxrB to elicit downstream regulatory effects **(Fig. S1A)**. We previously showed that the *vxrAB* locus itself is induced by PenG, and sought to establish its PenG-dependent regulon ^14^. Conducting the same RNAseq protocol, we thus explored how transcripts changed in a Δ*vxrAB* mutant background. We then compared Δ*vxrAB* regulon transcripts to the levels observed in WT strains and found that nearly all VxrAB regulated genes (as determined by our previous ChipSeq experiments ^14^) were differentially regulated by PenG **(Fig. S1B)**. Thus, the VxrAB regulon is activated by PenG exposure.

### Central metabolism gene expression changes in response to PenG exposure

Previous studies suggested that TCA cycle genes are induced as a consequence of exposure to b-lactam antibiotics ^24^. Our analysis here revealed that genes involved in central carbon metabolism and the TCA cycle experienced minor dynamic flux over time (Fig. 1B). Some genes, like *vc1304* and *vc1141*, (coding for fumarate hydratase and isocitrate dehydrogenase respectively), were slowly reduced in expression, reaching a 2.8- fold reduction by 90 minutes of exposure. In contrast, genes coding for the fumarate reductase complex (*frdABCD)*, employed during anaerobic respiration, were modestly (2-fold) increased at 90 minutes. Gene coding for earlier steps in the TCA cycle also displayed an increase in expression, such as *vc2084* and *vc2085* (~4-fold change increase in early penG exposure), coding for the succinyl-CoA synthetase complex; genes *vc2086* and *vc2087*, creating 2-oxoglutarate dehydrogenase; and genes *sdhCDAB* (*vc2088-vc2091)*, which make up the succinate dehydrogenase complex. The upregulation of late-TCA cycle enzymes could indicate a demand for more energy and/or reducing equivalents. The TCA cycle and subsequent respiration creates the most ATP molecules compared to other metabolic pathways, and it seems plausible that the cell attempts to optimize ATP generation given that β-lactam tolerance relies heavily on energy-intensive damage repair pathways ^14,16^. These results could also indicate a resource allocation requirement, in which high amounts of succinate or fumarate are needed for the biosynthesis of other metabolites, perhaps amino acids or nucleotide production.

We next focused on cell signaling pathways, including two-component systems (TCS), which are the primary cell signaling means of bacteria. Cell signaling pathways consist of kinases, which respond to diverse signals, and (sometimes through intermediary proteins) associated response-regulators, which activate specific regulons. The kinases are often autoregulated, and activated by antibiotic stress conditions, and we thus focused specifically on *V. cholerae*’s known TCS to uncover novel antibiotic-responsive signaling systems (Fig. 1C). Interestingly, the response regulator *vc1638*, encoding a homolog of PhoP, was induced nearly 16-fold (even higher than the primary cell wall damage response VxrAB). The PhoPQ TCS has been well-characterized in Enterobacterales, where it is induced by antimicrobial peptides, acidic conditions, and magnesium limitation^25^. We recently found that it is also activated by b-lactams and promotes carbapenem tolerance in *Enterobacter cloacae,* likely via OM stabilization upon L-amino-arabinose addition ^9^. Its role in *V. cholerae* is currently poorly characterized but the system has been implicated in polymyxin resistance via outer membrane modifications ^26^, alluding to a role similar to PhoPQ in *Enterobacterales*. The other notable change in two-component systems was a reduction in *citAB* expression level. This system regulates anaerobic citrate utilization in *V. cholerae* ^27^ and was strongly downregulated in the presence of PenG. CitAB has been shown to integrate multiple signals, including anaerobiosis, presence of citrate, and glucose levels (through catabolite repression) ^27^, and future work will focus on the role of this two-component system in the response to PenG.

Consistent with historical data ^28–30^, we also found many heat shock response ^31^ genes to be significantly upregulated in response to penicillin treatment (Fig. 1D). We previously showed that PenG exposure causes accumulation of oxidized proteins in *V. cholerae* ^14^, and we propose that these likely misfolded proteins induce the heat shock response upon PenG exposure.

### Nucleotide synthesis pathways are upregulated upon PenG exposure.

**Figure 2:**
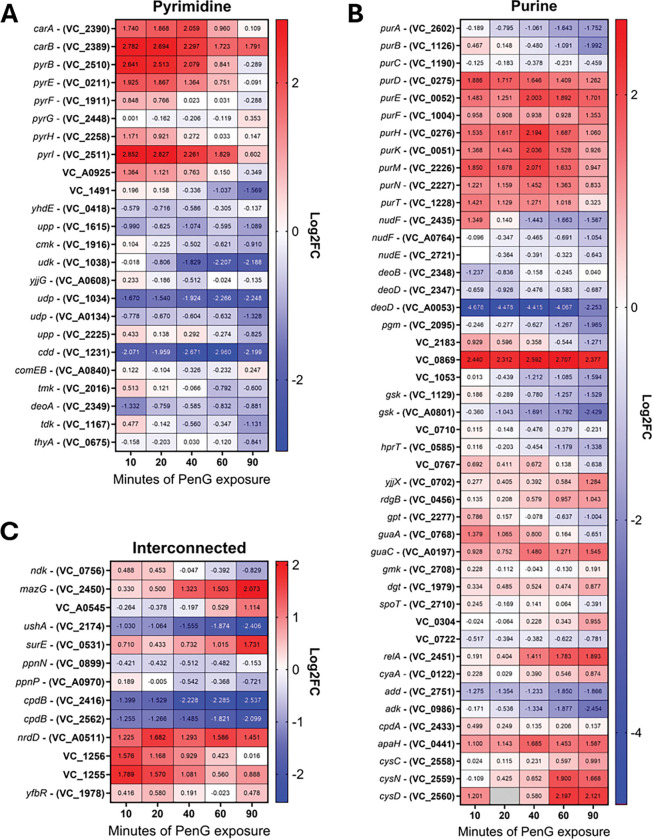
Transcriptomic changes from penicillin exposure over time. A) Pyrimidine biosynthesis pathway. B) Purine biosynthesis pathway. C) genes involved in both nucleotide biosynthetic pathways. See methods for details. Cut-off is p-value < 0.05, with raw log2 FC presented.

We noticed a large number of genes related to nucleotide biosynthesis in our transcriptomics dataset. Most of these genes were upregulated in response to penicillin treatment, particularly at early time points (Fig. 2A–C). Both purine and pyrimidine pathways were induced, with the majority of *de novo* synthesis genes being upregulated in response to PenG. This included the *purA-T* genes (*de novo* purine biosynthesis), as well as *pyrBEFGH* (de novo pyrimidine synthesis) and *carAB,* whose products convert L-glutamine to carbamoyl phosphate on the way towards pyrimidine biosynthesis ^32,33^ (Fig. 2AB). In contrast to these largely upregulated synthesis pathways, numerous genes coding for proteins involved in nucleotide homeostasis were markedly downregulated. These included nucleotide salvage pathways like *deoD – gsk* (coding for purine nucleotide phosphorylase and inosine kinase respectively); *cdd*, coding for cytidine deaminase; *udp*, coding for uridine phosphorylase; and UDP sugar hydrolases like *ushA* (*vc2174).* Thus, PenG exposure resulted in upregulation of nucleotide synthesis functions, and downregulation of nucleotide “recycling”, perhaps reflecting that a physiological need for preserving nucleotides has been sensed.

Interestingly, the stringent response gene *relA* was also upregulated. This enzyme creates (p)ppGpp, a nucleotide-based second messenger with the capabilities to reprogram cell physiology. In other bacteria, RelA plays vital roles in stress survival (e.g., amino acid starvation), virulence and antibiotic tolerance ^34,35^. Indeed, in a special case, *Staphyloccus aureus* upregulates two ppGpp synthetases RelQ and RelP in response to b-lactam exposure, promoting tolerance ^36^. This prompted us to test whether the stringent response also confers tolerance to *V. cholerae* spheroplasts. To test the effects of (p)ppGpp on tolerance levels, we created a deletion mutant that lacks production of (p)ppGpp altogether (Δ*relA*Δ*spoT*Δ*relV*, phenotypically confirmed via necessity of external supplementation of casamino acids during growth in minimal medium, **Fig. S2A**). However, this mutant did not yield any observed defects in antibiotic tolerance, nor resistance (**Fig. S2B-D).** This suggests that the stringent response does not play a role in PenG tolerance in *V. cholerae* under these conditions.

### Metabolomics highlight metabolite flux during PenG exposure

**Figure 3:**
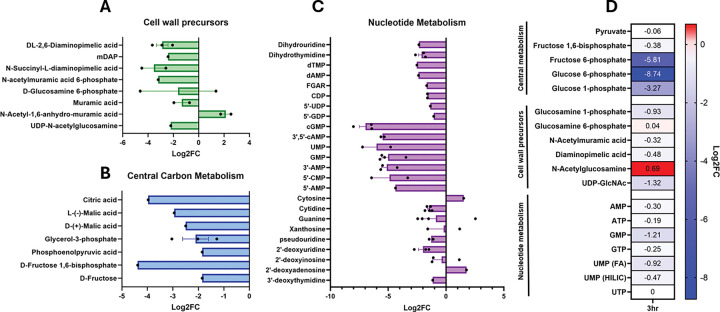
Metabolomics of penicillin treated cells. A) metabolites involved in central carbon metabolism. B) metabolites involved in the bacterial cell wall. C) metabolites involved in nucleotide metabolism. Data present mean fold change between the different detection modes of untargeted metabolomics and error bars represent SD. D) metabolites averaged and normalized to the non-treated condition of targeted metabolomics. See method for details.

To gain an understanding of how transcriptional changes correlate with cellular physiology, we next sought to identify changes in global metabolism associated with PenG exposure. To start with an unbiased approach, we conducted untargeted metabolomics. Following the same growth conditions previously used for the RNAseq experiment, we took samples at two different time points of penicillin treatment, 1 hour and 3 hours. Metabolites were extracted using ice cold methanol, and samples were analyzed using LC-MS (see Methods and Materials for details). Four different columns were used (HILIC positive/negative, and C18 positive/negative) to account for charge variations amongst the complete set of metabolites. After analysis of over 1,000 metabolites, we arranged the datasets for significant log2 changes, with each dot representing detection on unique columns (**Table S1** for full analysis). Displayed here are three subsets of the significant hits in relevant pathways (Fig. 3A–C).

Metabolites associated with cell wall synthesis exhibited a general decrease in abundance, consistent with previous results showing depletion of cell wall precursors in β-lactam-treated cells ^37,38^ due to “futile cycling” ^18^ (Fig. 3A). In contrast, abundance of one cell wall metabolite, *N*-acetyl-1,6-anhydro-muramic acid, was elevated. This metabolite is the product of peptidoglycan turnover, and confirms that β-lactam exposure elevates cell-wall recycling due to enhanced cell wall degradation ^11,39^; it thus serves as an internal control that our untargeted metabolomics revealed physiologically relevant metabolite changes. Interestingly, one isoform of D-glucosamine-6P increased in abundance, while the other isoform decreased (**Table S1**). We hypothesize that these two “isoforms” are in fact glucosamine-6P and glucosamine-1P, which we found to be differentially abundant in our targeted experiments (see below).

One major pathway impacted by penicillin treatment was central carbon metabolism, specifically glycolysis. (Fig. 3B). Abundance of metabolites within the entire EMP pathway, from glucose-6-phosphate to pyruvate, decreased upon PenG treatment. Some metabolites were reduced by 4-fold over control while others, like fructose-1,6-bisphosphate and citric acid, experienced an over 8-fold decrease. Pentose phosphate pathway intermediates were also reduced (**Fig. S3**). These observations are consistent with rerouting of central carbon flux upon PenG exposure.

Lastly, nucleotide metabolites, both purines and pyrimidines and their pathway intermediates, were dramatically depleted in PenG-treated cells, especially the monophosphates (Fig. 3C). Interestingly, ATP levels appeared to be unchanged, which is different from β-lactam exposure in non-tolerant organisms like *E. coli* and *Mycobacteroides abscessus*, where ATP levels are at least transiently elevated in response to b-lactams. Elevated ATP has been proposed to be associated with respiratory burst and consequently additional killing by reactive oxygen species^17,40–42^. The depletion of diverse nucleotides we observed in our metabolomics dataset is consistent with the upregulation of nucleotide synthesis genes observed in our RNAseq dataset, as the transcriptional changes likely reflect the cell’s nucleotide starvation response, e.g. through PurR (purines) and other complex regulatory pathways ^43^. We also observed a significant reduction in pentose phosphate pathway intermediates, like ribulose-5-phosphate (Ru5P) and erythrose-4-phosphate (**Fig. S3**). Our integrated -omics data thus indicate that penicillin treatment may ultimately starve the cell for energy and/or building blocks for diverse biosynthesis pathways.

To further interrogate our untargeted metabolomics results, we conducted targeted metabolomics on cells treated with penicillin for 3 hours and quantified select indicator metabolites that reflect cell wall intermediates, glycolysis intermediates, and nucleotides. We subjected the extracted metabolites to LC-MS using a HILIC probe and compared the peaks to known standards. We normalized the average of 4 biological replicates to the non-treated condition and assembled heatmaps representing altered metabolites (Fig. 3D). First, we quantified the abundance of key glycolytic intermediates glucose-1P, glucose-6P, fructose-6P, fructose-1,6-BP, and pyruvate. After 3 hours of penicillin exposure, we observed a reduction in all glycolytic metabolites, but the most extreme change was observed in glucose-6P and fructose-6P, which were reduced by 256-fold and 64-fold, respectively (Fig. 3D). Thus, PenG exposure dramatically alters glycolytic flux in tolerant spheroplasts, as suggested by our untargeted metabolomics. Glucose-6P connects to multiple metabolic pathways, and fructose-6P feeds directly into PG synthesis; the observed reduction in abundance could be indicative of altered flux from glycolysis via F6P (GlmS) into futile PG synthesis. We thus asked whether the depletion had physiological consequences, i.e. whether the depletion of G6P was harmful or protective to tolerant cells. Indeed, in the closely related L-Forms ^44,45^, addition of glucose is detrimental, causing enhanced respiration and concomitant generation of reactive oxygen species ^46^. To test whether spheroplasts exhibited a similar physiology, we supplemented our growth medium with glycolytic intermediates and conducted time-dependent killing (**Fig. S4A**). Pyruvate, fructose, and glucose at 0.2% did not affect PenG killing, suggesting that despite rapid depletion of intermediates, the ability to conduct glycolysis is neither limiting nor detrimental for *V. cholerae* spheroplasts.

We also used targeted metabolomics to confirm a reduction in UDP-GlcNAc, as well as other important cell wall precursors, like Diaminopimelic acid (DAP), N-Acetylmuramic acid, and glucosamine-1P (Fig. 3D). This is an expected result due to redirection of carbon flux into futile PG synthesis and is consistent with previous observation in diverse bacteria showing depletion of cell wall precursors upon β-lactam exposure ^18^. Lastly, we confirmed that nucleotides decreased upon penicillin treatment (Fig. 3D). GMP experienced the most pronounced reduction at 2.3-fold. UMP levels also dropped, possibly from the demand for UDP carriers involved in trying to repair the cell wall during penicillin treatment. Thus, our targeted metabolomics confirmed that central carbon metabolism, cell wall precursor levels and nucleotide homeostasis are affected by b-lactam treatment, as indicated by our untargeted metabolomics.

### Disruption of nucleotide homeostasis potentiates killing by PenG.

**Figure 4:**
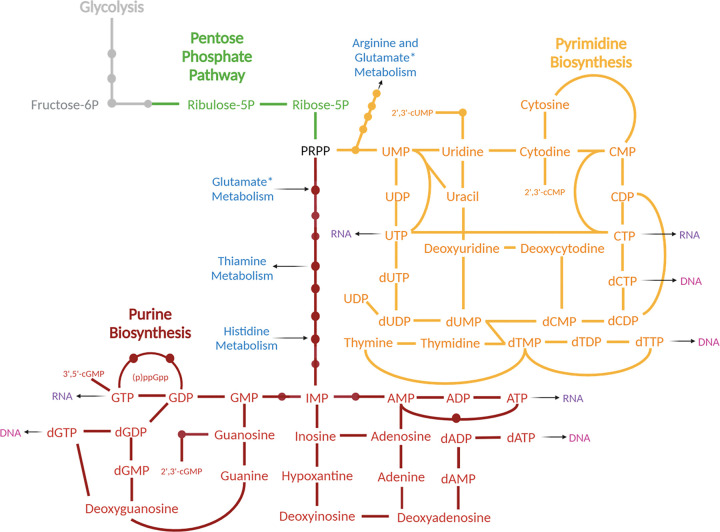
Nucleotide biosynthesis map. Major metabolites are presented with lines indicating connecting reactions. From central metabolism (gray) to the pentose phosphate pathway (green), the metabolites then get funneled into either the purine (red) or pyrimidine (orange) pathways. Relevant amino acid branch points are listed in blue, and genetic material is denoted in purple and pink.

**Figure 5:**
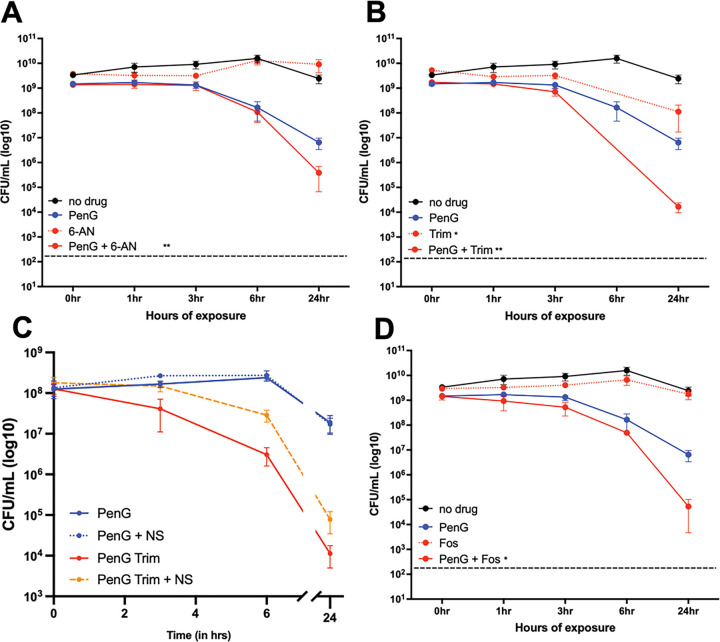
Nucleotide synthesis and PG precursor inhibition synergize with PenG A) CFU/mL were calculated over time with (+) and without (−) penG, and/or 6-aminonicotinamide (6-AN). B) CFU/mL were calculated over time with (+) and without (−) penG, and/or trimethoprim (Trim, 50 μg/mL, 60 × MIC). C) Cells were treated as described for B), but with or without addition of nucleosides (NS) D) CFU/mL were calculated over time with (+) and without (−) penG, and/or fosfomycin (Fosfo, 100 μg/mL, 4 × MIC). Panel A, B and D are means of at least 6 independent biological replicates. Panel C represents the means of 3 biological replicates. Statistical significance determined by unpaired t-test with Welch’s Corrections (* = p<0.05, ** = p<0.01).

Next, we sought to probe the potential for synergistic action with PenG of the pathways uncovered in our combined transcriptomics and metabolomics. To this end, we used chemical inhibitors and supplements designed to inhibit or boost different pathways. After growing cells to exponential phase, we treated our cells with 100μg/mL PenG for 1 hour. We then added the indicated additional chemicals and plated for cell viability over time. We found that inhibiting the pentose phosphate pathway (through treatment with 6-aminonicotinamide ^47^) exacerbated the killing effect of penicillin after 24 hours (Fig. 5A). The pentose phosphate pathway supplies a key precursor for nucleotide synthesis, PRPP (phosphoribosyl pyrophosphate) (see Fig. 4). Our results therefore indicate that PenG-treated cells are indeed starved for nucleotides, and that proper nucleotide homeostasis is essential for optimal survival of b-lactam exposure. These results add support to our -omics data implicating nucleotide levels as an important aspect of b-lactam susceptibility, as well as finding novel ways to decrease antibiotic tolerance in *V. cholerae*.

Seeking to unravel the connection between nucleotides and tolerance further, we challenged *V. cholerae* with both PenG and trimethoprim (trim), which blocks the conversion of dihydrofolate to tetrahydrofolate (ultimately resulting in purine and pyrimidine starvation) and measured survival (Fig. 5B). After 24 hours, trim by itself (50 μg/mL, 60 × MIC) reduced viability by 10-fold, while PenG caused the typically observed 50-fold killing (at 100 μg/mL, 10 × MIC). In combination, however, we observed a 10^5^-fold reduction in viability, demonstrating synergistic killing between trim and PenG.

We reasoned that if nucleotide pools are limiting in PenG-treated cells, external addition of nucleotides should rescue PenG-mediated lethality. We tested a variety of nucleotides, as well as nucleotide analogs with documented inhibitory effects ^48–51^. These supplementations did not cause any observable changes to PenG tolerance (**Fig. S4B-C**). Many bacteria import nucleosides at a higher rate than nucleotides ^33,52^, and we thus used a nucleoside mix, treated with and without PenG, and plated for CFU/mL. Interestingly, this supplementation did not have any observable effects on tolerance either (Fig. 5C, **Fig. S4D-E**). Lastly, we turned to our synergy experiment with trimethoprim. Under these conditions, nucleoside supplementation significantly reduced synergistic killing (Fig. 5C), but did not restore viability observed with PenG single treatment. In aggregate, these experiments suggest that i) nucleosides are imported by *V. cholerae* during antibiotic exposure, ii) nucleoside levels are not limiting for spheroplasts survival (perhaps due to the observed upregulation of *de novo* synthesis pathways) and iii) nucleosides can become limiting in spheroplasts when their synthesis is additionally suppressed. In an attempt to genetically manipulate the purine biosynthesis pathway, we next deleted the nucleotide starvation regulator PurR. We did not observe any defects in PenG tolerance nor resistance (**Fig. S5A-B**). This was surprising, as other studies have found altered antibiotic efficacy of a *purR* deletion mutant in the presence of β-lactam antibiotics ^51,53–55^. It is possible that *V. cholerae’s* regulation of nucleotide starvation does not mimic what has been established in well-studied model organisms, and further work is needed to uncover the regulatory effects of PurR in this system.

**Figure 6:**
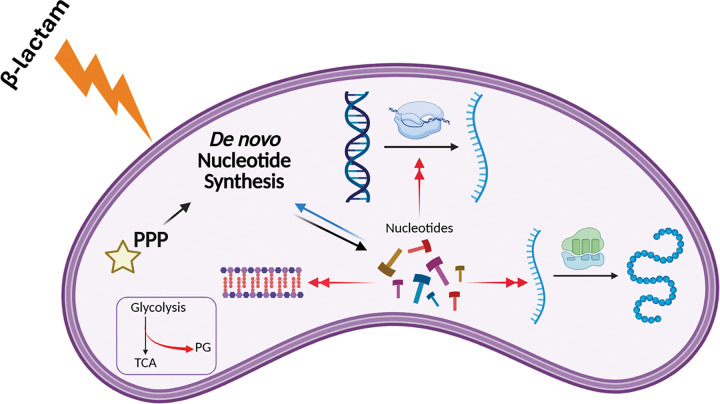
Model of nucleotide involvement in tolerance. Upon β-lactam exposure, the nucleotide pool is placed under stress, from processes like transcription, translation, and cell wall repair. This causes *de novo* biosynthesis of nucleotides to be activated. Inhibiting the pentose phosphate pathway (PPP), which feeds *de novo*biosynthesis, through the exposure of 6-aminonicotinamide, the cell suffers more from penicillin exposure.

Lastly, we also reasoned that the depletion of PG precursors should make tolerant cells particularly susceptible to inhibition of PG precursor synthesis. We thus exposed cells to a combination of PenG (100 μg/mL, 10 × MIC) and Fosfomycin (100 μg/mL, 4 × MIC). Fosfomycin inhibits MurA, catalyzing an early step in PG precursor synthesis. Interestingly, fosfomycin by itself, despite ultimately causing cell wall degradation like PenG ^19^, did not result in any reduction in viability. This perhaps reflects that PenG does indeed cause death through more than just cell wall degradation, i.e. the additional killing is due to futile cycling and other metabolic demands. However, in combination, PenG and fosfomycin caused a dramatic, 10^5^-fold reduction in plating efficiency, demonstrating strong synergy between PenG and precursor synthesis inhibition (Fig. 5C). We conclude that PG precursors are limiting in tolerant cells (see proposed model in Fig. 6).

## Discussion

Antibiotic tolerance is an understudied contributor to antibiotic treatment failure. The mechanisms and physiology of tolerance have only begun to be unraveled. In this work, we dissected the alterations in gene expression and metabolic flux associated with antibiotic tolerance. Using a multi-omics tool set and a hypertolerant model organism, we identified large genetic and metabolic perturbations in multiple key categories including cell wall biosynthesis, heat shock response, the TCA cycle, and nucleotide metabolism. Pathways that are altered in tolerant cells could serve as targets for future exploration of antimicrobial adjuvants, which we have begun in this study by showing that disturbing nucleotide homeostasis strongly synergizes with a β-lactam antibiotic against tolerant *V. cholerae*.

A key unanswered question about spheroplast-mediated tolerance is how these bacteria mitigate the dramatic damage sustained due to loss of their cell wall. It has become clear in recent years that antibiotics cause disturbances in cellular physiology that go beyond the simple direct consequences of target inhibition. Indeed, we and others have previously measured a marked accumulation of reactive oxygen species in response to β-lactam exposure, likely due to dysregulation of the electron transport chain, though the degree to which this contributes to killing is unclear ^14,56,57^. Additionally, data from *E. coli* and *Streptoccus pneumoniae* demonstrate that β-lactams induce “futile cycling”, i.e. the constant generation and degradation of uncrosslinked PG strands, which ultimately results in poorly-understood energy and redox imbalance, thereby contributing to cell death and/or growth arrest^17,18^. However, spheroplasts do not appreciably die over extended time periods, indicating that they possess efficient repair processes that mitigate both ROS production and negative consequences of futile cycling. Our data shed more light on how this might be accomplished. For example, one of the immediate consequences of ROS accumulation is the production of oxidized (e.g. likely dysfunctional) proteins^12^. In our transcriptomics dataset we show that cells respond to this by highly upregulating heat shock proteins, which might help remove or refold non-functional proteins. Our metabolomics experiment shows clear signatures of futile cycling, i.e. depletion of cell wall precursors and concomitant rerouting of central carbon metabolism away from glycolysis. At the same time, ATP homeostasis does not appear to be disturbed, indicating that spheroplasts are perfectly capable of buffering the energy demand that should be associated with changed metabolism. The observation that nucleotides are depleted in spheroplasts, concomitant with upregulation of nucleotide synthesis pathways can be interpreted as an indirect consequence of futile cycling. Rerouting of central carbon metabolism into futile cell wall synthesis could both deplete precursors for the pentose phosphate pathway (which ultimately generates nucleotide precursors) and also increase the use of nucleotides involved in activation of precursors, e.g. UDP-GlcNAc. The additional upregulation of stress responses (like the VxrAB cell envelope stress system) can be expected to put additional demand on ATP and GTP due to enhanced anabolic reactions. It is interesting in this context that the triphosphate form of nucleotides (ATP and GTP) themselves do not seem to be affected. This may indicate that nucleotide diphophosphate kinases have low K_d_ values and can operate optimally even in dramatically reduced substrate concentrations, explaining perhaps why this energy demand does not by itself have lethal consequences in tolerant spheroplasts.

On a broader note, our data uncover that spheroplasts appear to be in a physiological state that is distinct from susceptible bacteria or even the closely related L-Forms ^44,45^. For example, we found that the ability to generate ppGpp (a well-characterized facilitator of tolerance in persisters ^58^) does not contribute to tolerance in spheroplasts. In contrast to L-form survival ^46^ and *E. coli* resistance to killing ^59,60^, ROS levels do not seem to be limiting for spheroplast survival, and addition of glucose does not kill these cells. Also, unlike *E. coli* treated with β-lactams, we do not observe clear increases in ATP levels, protein synthesis or accumulation of glycolytic intermediates and cell wall precursors (in fact we observed the opposite). However, the connection between nucleotide homeostasis and b-lactam susceptibility is not without precedent, suggesting broad conservation: In *S. aureus*, mutations in the pentose phosphate pathway reduce oxacillin-induced lysis, though this is thought to be due to surface alterations and/or slow growth ^61,62^. In *B. subtilis,* nucleotide homeostasis is tied to cefuroxime resistance via mutations in RNA polymerase^63^. Overall, a somewhat inconclusive body of work has emerged on the role of nucleotide synthesis on tolerance, persistence, and resistance ^51^. This inconclusiveness might be caused by differential effects that the underlying physiology has on various forms of tolerance vs. various forms of resistance. Our results here now shed light onto the complex and interconnected response to β-lactam antibiotics in a hypertolerant bacterium. Our data also indicate novel points of vulnerability to devise new avenues for antibiotic adjuvants.

## Methods

### Strain construction

The wild-type strain used in this study is *V. cholerae* El Tor strain N16961 ^64^, and the Δ*vxrAB* strain was constructed in a prior study^16^. Deletion constructs were made using pTOX ^65^ . In short, 500bp upstream and downstream of the gene were PCR amplified using the relevant primers listed in **Table S2**. The fragments were then simultaneously ligated and inserted into the EcoRV-digested vector using Gibson Assembly and heat transformed into DH5α competent *E. coli* cells followed by selection on LB agar with 0.2% glucose (w/v) and 20μg/mL chloramphenicol. Plasmids were extracted using Omega Miniprep kit and verified via Sanger Sequencing. Plasmids were then heat transformed into conjugative MFDλpir and conjugated into WT N16961 following the steps outlined below.

Conjugation into WT N16961 went as follows: 100μL of donor and recipient were mixed together, spotted on LB agar with 60μM DAP for MFD, and left to incubate for 4 hours at 37°C. The inoculum was then scraped and spread on LB agar with 100μg/mL streptomycin and 20μg/mL chloramphenicol and 0.2% glucose (w/v) (pTOX) and incubated overnight at 30°C. Transconjugants were single-colony purified and then spread onto counter-selection plates: minimal media agar with 2% rhamnose (w/v) (pTOX). Plates were incubated overnight at 30°C. Colonies were screened for the correct deletion using primers in **Table S2.** All strains were additionally verified with whole genome sequence.

The *relV* gene was deleted in a Δ*relA*Δ*spoT* background (gift from the Waldor lab) using the allele exchange plasmid pCVD442. In brief, homology regions upstream and downstream of relV were amplified using primers TDP1112/1113 and TDP 1114/1115 and cloned into SmaI-digested pCVD442 using isothermal assembly. Successful clones (sequence verified) were conjugated into the Δ*relAΔspoT* strain using the donor strain SM10. Post-conjugation, transconjugant single crossover colonies were purified on strep (200μg/mL)/carbenicillin (100μg/mL). Single colonies were then spread on LB + strep + 10%sucrose, followed by incubation at 30°C overnight.Successful mutants were verified via colony PCR (using primers TDP 1110/1111).

### Bacterial Growth Conditions

*V. cholerae* was grown in Luria-Bertani (also called lysogeny broth) (LB) medium (for a 1L bottle, 10g Casein peptone, 5g yeast extract, 10g NaCl, and 12g agar, unless otherwise indicated. All premade from Fischer Bioreagents) at 30°C. 200μg/mL of streptomycin was also added (N16961 is streptomycin resistant). M9 minimal media was made at 1L bottle, 15g agar, 200mL 5x M9 salts (1L bottle contains 35g Na_2_HPO_4_•7H_2_O, 15g KH_2_PO_4_, 2.5g NaCl, 5g NH_4_Cl), 0.5mL 1M MgSO_4_, 0.1mL 1M CaCl_2_, 1mL FeCl_3_/citric acid, 10mL glucose (20% w/v). All samples were grown overnight at 30°C and experimented at 37°C.

### Cell culture condition for RNA-Seq

Wild-type and mutant strains were from an isolated single colony and grown in LB medium containing 200μg/mL streptomycin overnight at 30°C. 2mL of the culture was then added to 250mL LB + 200μg/mL streptomycin in a 1L flask and left to grow until mid- to late exponential phase (OD600 = 0.4 – 0.5). 30mL of exponential phase cultures were pelleted after treatment of (+) / (−) 100μg/mL PenG as indicated time points (0, 10, 20, 40, 60, 90 minutes). RNA was isolated by using TRIzol reagent (Ambion) and DNA was removed using the Turbo DNase kit (Ambion). RNA samples were pooled as pool 1 (first set of biological triplicates and technical replicate 1) and pool 2 (second set of biological triplicates and technical replicate 2). Following cDNA synthesis, samples were subjected to high-throughput sequencing. The RNAseq data was first uploaded to the Galaxy web platform (https://usegalaxy.org) for quality control and trimming of the raw sequencing reads. Next the trimmed reads were aligned to the *Vibrio cholerae* N16961 reference genome (from NCBI: GCF_000006745.1) and htseq-count was used to calculate the number of reads mapping to each feature. The count files were then used in R to perform differential expression analysis using DESeq2. The resulting log2FoldChange values were then used in GraphPad Prism 9 to generate heatmaps for visual representation of the data.

### Cell culture conditions for metabolomics

Three or four biological replicates were grown in LB overnight at 30°C (for untargeted and targeted respectively). 2mL of culture was added to 250mL of LB in a 1L flask and incubated 1.5 hours at 37°C, till OD600 was between 0.4–0.6. (+) / (−) 100μg/mL PenG was added to the flasks and left to incubate an additional 1 or 3 hours. After treatment, 2mL of sample were taken per condition and pelleted at 7000rpm for 2 min. The supernatant was removed, and the cell material pellet was flash frozen with liquid nitrogen. 200μL of cold 80% methanol was added to the pellets. Pellets were stored at −80°C.

Untargeted metabolomics were sent to the Cornell BRC facility for preparation and analysis. In summary this was their procedure: First that samples were vortexed for 5 min on vortex mixer (1800 rpm/5minutes), pellet disintegrated and is mixed thoroughly in extraction solvent. Cells were then sonicated (in Ultrasonic Bath) for 20 min. Cells were then vortexed for 10 seconds to evenly resuspend. Immediately removed normalized amount (based on number of proteins in each sample). Added 80% Methanol to make final vol = 600μl in each sample. Incubate 60min/4°C. Centrifuged 16,000g/10mins/4°C, removed supernatant, vol = 250μl X2 into separate 1.5 ml Eppendorf tubes (2 aliquots -one for each for HILIC & C18 analysis). Dried to dryness in speed vac. Store at −20C until ready to analyze. Reconstitute the dried extract in 200μL ACN 20%, 0.1% FA (30μL) for C18 and ACN 20% for HILIC. Sonicated the solution for about 5 min and then transferred into LCMS vial, 10μl for QC for each group and keep the rest at −20°C.For assessing the LCMS machine performance, two QA samples containing 15 standard compounds were run before and after real samples respectively. For normalizing the chromatograms, a global Quality Control sample containing all groups was run twice at the beginning of the sequence, at the end, and every 6 samples, and for compound identification, each of three group QCs (0, 1, 3 hours) was run twice by MS2. Global Quality Control sample is a pool of all the samples. Group QC sample is a pool of all 6 samples within the time group.

Compound discoverer was used for untargeted metabolomics analysis, comparing the m/z in the samples with different databases: Chemspider, bioCyc, HMDB, SMDPD, Lipidmaps, Mzcloud. C18 LC-MS summary: 589 and 441 annotated metabolites were identified following CD search and filtering for positive ion and negative ion mode respectively. After removing the background and redundancy, 554 and 413 unique metabolites were annotated, in which 318 and 218 metabolites were identified by MS/MS for preferred ion. Those metabolites with MS2 should be more confident. HILIC LC-MS summary: 787 and 505 annotated metabolites were identified following CD search and filtering for positive ion and negative ion mode respectively. After removing the background and redundancy, 760 and 502 unique metabolites were annotated, in which 696 and 469 metabolites were identified by MS/MS for preferred ion. Presented are those with a > 2-fold change and an adjusted p-value < 0.05. Those identified metabolites with MS2 should be more confident (see excel attached), Note: Same name of metabolites with retention times >0.3min were manually labeled by original name iso, for which more than 2 isoforms labeled by iso1…4 that are considered as unique isoforms. For targeted metabolomics, HILIC protocol and analysis was conducted following ^66^. Heatmaps were generated using Prism, with averaged peak heights normalized to the control ((−) PenG) condition.

### Synergy Killing Assay

WT *Vibrio cholerae* cultures were grown overnight in 5mL LB broth at 30°C. The following morning, they were diluted 1:100 into fresh LB and grown at 37°C for 1.5 hours. (+) / (−) 100μg/mL PenG was then added for 1 hour. Other chemicals were then added at the following concentrations: 100μg/mL 6-aminonicotinamide (6-AN); 100μg/mL fosfomycin (fosfo); 50μg/mL trimethoprim (trim) (others designated in figure legends). The nucleotide mix was purchased from MilliporeSigma^™^ EmbryoMax^™^ Nucleosides (100X) and contained nucleotides at the following concentrations; Cytidine: 0.73g/L; Guanosine: 0.85g/L; Uridine: 0.73g/L; Adenosine: 0.8g/L; Thymidine: 0.24g/L. We used a 2x final concentration (50fold dilution of stock) for our assays. After chemical addition, 100μL samples were taken at 0, 1-, 3-, 6- (for some of the experiments), and 24-hour timepoints and serially diluted in 1xPBS. 5μL spots were plated on LB agar (or M9 minimal media supplemented with 0.2% glucose) and incubated overnight at 30°C (37°C for M9). CFU/mL were counted the following day.

## Data Availability

The sequencing data have been deposited in the National Center for Biotechnology Information’s Sequence Read Archive (SRA) under BioProject PRJNA1338477.
